# Comparison of postoperative atrial fibrillation after total coronary revascularization via left anterior thoracotomy and conventional median sternotomy coronary artery bypass grafting

**DOI:** 10.3389/fcvm.2025.1697113

**Published:** 2025-10-31

**Authors:** Sefa Sural, Vedat Aslan, Deniz Mutlu, Nurettin Yeral, Ozerdem Ozcaliskan, Gokhan Gokaslan

**Affiliations:** ^1^ Department of Cardiology, Faculty of Medicine, Istinye University, Sariyer, Türkiye; ^2^ Department of Cardiology, SUNY Downstate Health Sciences University, Brooklyn, NY, United States; ^3^ Department of Cardiology, Hatay Training and Research Hospital, Hatay, Türkiye; ^4^ Department of Cardiovascular Surgery, Özel Mersin Yenişehir Hospital, Mersin, Türkiye; ^5^ Department of Cardiovascular Surgery, Özel Gaziantep Anka Hospital, Gaziantep, Türkiye

**Keywords:** coronary artery bypass, postoperative atrial fibrillation, left anterior thoracotomy, TCRAT, median sternotomy

## Abstract

Postoperative atrial fibrillation (POAF) is the most common arrhythmia that occurs after coronary artery bypass grafting (CABG), contributing to increased mortality, morbidity, longer hospital stays, and higher healthcare costs. Total coronary revascularization via anterior thoracotomy (TCRAT) has recently emerged as a minimally invasive alternative to the traditional median sternotomy (MS). In this multicenter retrospective cohort study, 424 patients who underwent elective CABG between 1 January 2022 and 31 December 2024 at three centers were analyzed. Of these, 221 patients received TCRAT and 203 underwent MS. To minimize baseline differences, a propensity score matching of 1:1 was performed based on age, sex, left ventricular ejection fraction (LVEF), left atrial diameter, CHA_2_DS_2_-VASc score, systolic pulmonary artery pressure, and baseline β-blocker use. POAF was defined as an episode of atrial fibrillation lasting a minimum of 5 min and confirmed by electrocardiography. Independent risk factors were identified using a multivariate logistic regression analysis. The rate of incidence of POAF was 16.7% in the TCRAT group and 25.1% in the MS group (*p* = 0.045). After matching, a multivariate analysis showed that the traditional surgical approach, MS, was an independent risk factor for POAF [odds ratio (OR), 6.12; 95% confidence interval (CI), 2.48–15.09; *p* < 0.001]. Advanced age (OR 1.04, *p* = 0.019), reduced LVEF (OR 0.95, *p* = 0.019), longer cross-clamp time (OR 1.07, *p* < 0.001), higher blood transfusion requirements (OR 1.48, *p* = 0.004), and diabetes (OR 1.91, *p* = 0.048) were all identified as independent predictors. Despite longer operative and cross-clamp times, TCRAT was associated with a lower incidence of POAF compared with MS.

## 1 Introduction

Atrial fibrillation (AF) is among the most common arrhythmias observed after the performance of coronary artery bypass grafting (CABG). The overall rate of incidence of postoperative atrial fibrillation (POAF) is approximately 30% across all types of cardiac surgeries, while in patients undergoing isolated CABG, the rate is approximately 20%. In patients undergoing valve surgeries, however, the rate of incidence of POAF can reach as high as 50% (1, 2). Regardless of the surgery type, POAF is linked to extended hospital stays, a higher risk of adverse cardiovascular events, and greater mortality. In addition, POAF increases healthcare costs and the long-term risk of stroke (3, 4).

Traditionally, surgical treatment for coronary artery disease has been performed safely and effectively by employing procedures such as median sternotomy (MS) and cardiopulmonary bypass (CPB), and myocardial protection techniques (5). However, efforts to reduce the physical and psychological trauma associated with CABG and enhance the quality of life of patients have led surgeons to increasingly adopt less invasive methods (6).

In 2019, Babliak et al. introduced total coronary revascularization via anterior thoracotomy (TCRAT), an on-pump technique performed through a left mini-thoracotomy. Independent of vessel number, quality, or patient characteristics, TCRAT has demonstrated high versatility with the use of multiarterial grafting and is now considered an attractive alternative to conventional sternotomy (7, 8).

Although evidence supporting the short-term outcomes of TCRAT is growing, data on arrhythmic complications remain limited, and studies comparing the incidence of POAF between TCRAT and MS are rare. Therefore, the present study aims to compare the development of POAF following CABG performed via TCRAT versus MS and evaluate the factors associated with POAF.

## 2 Materials and methods

### 2.1 Study design and patient selection

This study was conducted as a retrospective cohort analysis. A total of 424 patients who underwent elective CABG at Mersin Medicalpark Hospital, Gaziantep ANKA Hospital, and Mersin Yenişehir Hospital were evaluated. Of these, 221 patients underwent TCRAT, while 203 underwent median sternotomy CABG (MS-CABG). To minimize the effect of the learning curve, the first 30 TCRAT cases per surgeon were excluded (157 cases from the first surgeon and 37 from the second). Therefore, only procedures performed between 1 January 2022 and 31 December 2024 were included in the analysis. To ensure temporal comparability, both TCRAT and MS-CABG procedures performed by the same surgeons during this period were included in the study cohort.

**Figure 1:**
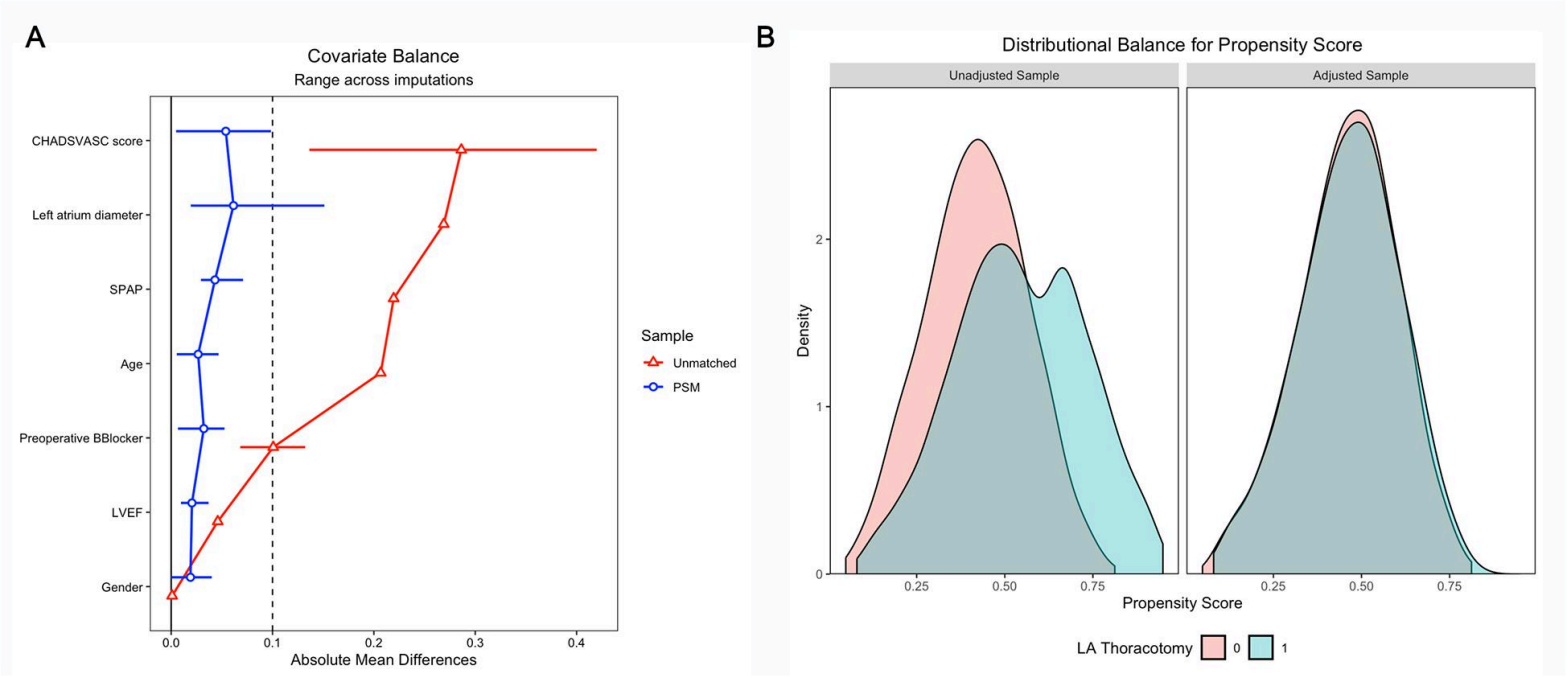
**(A)** Covariate balance plot demonstrating standardized mean differences before and after PSM. **(B)** Kernel density plot showing the distribution of propensity scores between TCRAT and MS groups after PSM. LVEF, left ventricular ejection fraction; PSM, propensity score matching; SPAP, systolic pulmonary artery pressure; MS, median sternotomy; TCRAT, total coronary revascularization via left anterior thoracotomy; LA thoracotomy, left anterior thoracotomy.

To reduce baseline imbalances between the groups, propensity score matching (PSM) was performed in a 1:1 ratio based on age, sex, and key clinical variables, including CHA_2_DS_2_-VASc score, left ventricular ejection fraction (LVEF), left atrial (LA) diameter, systolic pulmonary artery pressure (SPAP), and β-blocker use (Figure 1).

### 2.2 Surgical techniques

#### 2.2.1 TCRAT

Patients were placed in the supine position with approximately a 30° tilt and a pad under the left chest to facilitate dissection of the left internal mammary artery (LIMA) and cardiac exposure. Anesthesia was provided using standard cardiac techniques. A double-lumen endotracheal tube was inserted, and intraoperative hemodynamic monitoring with transesophageal echocardiography (TEE) was performed in all patients.

Femoral artery/vein cannulation (Biomedicus® 21–25 Fr, Medtronic, for venous cannulation and EOPA® 18–20 F, Medtronic, for arterial cannulation) was performed—most commonly through the right femoral artery—taking into account the atherosclerotic plaque burden of the iliac and femoral arteries on preoperative CT angiography. Jugular venous cannulation (Biomedicus® 15–17 Fr, Medtronic, Minneapolis, MN, USA) was routinely added. All cannulations were performed under TEE guidance. One patient was converted to open surgery because femoral venous cannulation could not be advanced.

In male patients, the incision was made through the fourth intercostal space from the sternocostal junction to the nipple line, while in female patients, an inframammary approach was used. After the initial learning curve (approximately after each surgeon's 30th TCRAT case), a muscle-sparing approach preserving the pectoralis muscle was adopted in all patients. Following entry into the thoracic cavity, single-lung ventilation was initiated and a retractor was placed. The LIMA was harvested in a skeletonized manner, while the saphenous vein graft (SVG) was prepared simultaneously. In suitable patients, the SVG was harvested endoscopically.

The pericardium was opened longitudinally, and the ascending aorta was dissected and encircled using a polyester tape. The third intercostal space was preferred for cross-clamping instead of the second. A mixture of blood and Del Nido cardioplegia solution was used for myocardial protection. For cardiac positioning, polyester tapes fixed to the inferior vena cava and left pulmonary vein were employed.

Distal anastomoses were constructed on the target vessels. Cardioplegia was administered after each bypass for control. During the left internal mammary artery to left anterior descending artery (LIMA–LAD) anastomosis, the patient was rewarmed, and hot cardioplegia was administered at the end of the procedure. After removal of the cross-clamp, sinus rhythm was observed, and proximal anastomoses were completed conventionally under partial clamping. Only the LIMA and saphenous vein were used as grafts.

#### 2.2.2 MS-CABG

Harvesting of the LIMA, radial artery (RA), and SVG was performed using standard techniques. The procedures were carried out with patients in the supine position.

Unlike the TCRAT approach, this patient group underwent surgery via the conventional MS approach. The LIMA was harvested as a pedicled graft under direct vision. After opening the pericardium, 400 U/kg intravenous heparin was administered. Central arterial cannulation (EOPA® 20–22 Fr, Medtronic) was performed through the ascending aorta and bicaval venous cannulation (32/40 Fr Bicaval, Medtronic) was performed through the right atrium. An antegrade cardioplegia cannula was also placed in the ascending aorta.

Normothermia was maintained during CPB. After aortic cross-clamping, the heart was arrested with antegrade cold blood cardioplegia, with repeated doses every 15–20 min.

Coronary anastomoses were performed using a continuous suture technique with 8-0 polypropylene. The LIMA was grafted *in situ* to the LAD. RA and/or SVG conduits were either connected to the LIMA in a T or Y configuration or directly anastomosed to the ascending aorta.

Complete revascularization was defined as the successful grafting of all angiographically significant coronary vessels with ≥70% stenosis and a vessel diameter ≥1.5 mm. This definition was applied equally to both types of surgical approaches (TCRAT and MS). No patients in either group received a radial artery graft.

### 2.3 Variables assessed

Preoperative data included age, sex, body mass index (BMI), comorbidities (hypertension, diabetes, chronic kidney disease, etc.), medications (particularly amiodarone, digoxin, beta-blockers), echocardiographic findings (including EF), CHA_2_DS_2_-VASc, and EuroSCORE II scores.

Intraoperative data included duration of surgery, CPB time, cross-clamp time (in the MS group), number and type of grafts used, and need for reexploration.

Postoperative data included time to extubation, length of ICU and hospital stay, and laboratory values prior to discharge. All patients were also evaluated for the occurrence of POAF. Outcomes of 30-day mortality and stroke were determined using hospital electronic medical records, 1-month outpatient follow-up visits, and telephone interviews when necessary to ensure complete 30-day ascertainment.

Complications included bleeding, reoperation, myocardial infarction, stroke, ventricular tachycardia, acute renal failure, infection, graft occlusion, cardiogenic shock, and mortality.

### 2.4 Definition and evaluation of POAF

Postoperative monitoring was conducted in the intensive care unit (ICU) and included daily complete blood count, biochemistry, chest x-ray, and continuous ECG monitoring. A 12-lead ECG was performed intraoperatively and during ICU stay. The primary endpoint was POAF, defined as an episode lasting ≥5 min within 30 days after surgery and confirmed by electrocardiography. After transfer to the ward, patients underwent 12-lead ECG twice daily during the first 24 h. Additional ECG recordings were obtained if symptoms or an irregular pulse was detected during physical examination. Patients with documented AF episodes were included in the POAF group.

### 2.5 Inclusion criteria

1. Underwent elective CABG with either TCRAT or MS between 2022 and 2024.
2. Presence of ≥70% stenosis in at least one coronary artery.

### 2.6 Exclusion criteria

1. Presence of a permanent pacemaker or implantable cardioverter-defibrillator (ICD).
2. Persistent AF or a history of paroxysmal AF (PAF) before CABG.
3. Use of amiodarone or digoxin before CABG.
4. Hemodynamically unstable patients or those with decompensated heart failure.
5. Patients who underwent concomitant valve surgery.
6. Patients undergoing redo CABG procedures.
7. Emergent surgeries.

### 2.7 Perioperative management and AF prophylaxis

All centers followed a uniform perioperative protocol to minimize the risk of atrial fibrillation. Electrolyte levels were optimized preoperatively and postoperatively (potassium >4.0 mmol/L, magnesium >2.0 mg/dL). β-blockers were continued when tolerated and resumed within 24 h after surgery in stable patients. Magnesium supplementation was routinely administered, while amiodarone prophylaxis was not used. Rate- and rhythm-control thresholds were consistent across centers, and all patients were continuously monitored in the ICU with daily ECGs until discharge.

### 2.8 Statistical analysis

Categorical variables were presented as absolute numbers and percentages and compared using the Chi-square or Fisher's exact test, as appropriate. Continuous variables were shown as mean ± standard deviation or median (interquartile range, IQR) and compared using the independent-sample t-test for normally distributed data and the Mann–Whitney *U* test for non-parametric data, as appropriate.

To handle incomplete data, five multiply imputed datasets were generated and analyzed using multiple imputation. Incomplete data were imputed using the fully conditional specification method, applying the default settings (*m* = 5) of the “mice” package in R (9). The percentage of missing data for the entire dataset was 6.2 (Supplementary Figure S1).

A PSM analysis was conducted to address potential confounding factors. Seven variables—age, gender, LVEF, LA diameter, CHA_2_DS_2_-VASc score, SPAP, and baseline β-blocker use—were included in the PSM analysis.

The analytical workflow followed a within-imputation matching strategy. Multiple imputation was performed first, followed by PSM conducted separately within each imputed dataset (MI → PSM → analysis → pooling). PSM was conducted using the “within” method instead of the “across” method because of its lower risk of bias (10). The nearest neighbor matching approach was applied using a 1:1 matching ratio and a caliper of 0.1. Matching was performed without replacement, and only observations within the region of common support were retained.

The distribution of propensity scores within each treatment group was visually inspected using a kernel density plot. Baseline and PSM-adjusted covariate balance was assessed through absolute standardized mean differences, all of which were below 0.1 (Supplementary Table S1).

Logistic regression was used to analyze the impact of the operation technique on atrial fibrillation development across the imputed datasets, and the results were pooled using the “pool()” function from the “mice” package (Supplementary Table S2). Variance Inflation Factor (VIF) was calculated to assess multicollinearity (Supplementary Table S3). Final multivariable logistic regression analysis was performed to identify independent predictors. Collinearity was evaluated using the variance inflation factor test, with values below 3 indicating no significant multicollinearity.

Model calibration was assessed using the Hosmer–Lemeshow goodness-of-fit test (*p* = 0.510), and discrimination was evaluated by using the area under the receiver operating characteristic (ROC) curve (AUC = 0.79) (Supplementary Figure S2). The linearity of continuous predictors was examined using logit plots. The final model included seven predictors and 78 POAF events, yielding an event-per-variable (EPV) ratio of 11.14, confirming adequate model stability.

All statistical analyses were performed using R Statistical Software, version 4.4.1 (R Foundation for Statistical Computing, Vienna, Austria). All tests were two-sided, with a *p*-value of <0.05 indicating statistical significance.

## 3 Results

A total of 424 patients undergoing elective CABG were included in the analysis (TCRAT: 221, MS: 203). As summarized in Table 1, patients in the MS group were significantly older than those in the TCRAT group (66.9 ± 10.9 vs. 64.7 ± 9.9 years, *p* = 0.027). There were no significant differences between the groups regarding sex distribution, BMI, or the prevalence of comorbidities such as hypertension, diabetes, chronic obstructive pulmonary disease (COPD), and hyperlipidemia. Mean EuroSCORE II values were also similar (Table 1).

**Table 1:** Baseline characteristics of the study population.

Variable	TCRAT (*n* = 221)	MS (*n* = 203)	*p*-value
Age (years), mean ± SD	64.7 ± 9.9	66.9 ± 10.9	0.027
Male, % (*n*)	73.3 (162)	73.4 (149)	1.000
BMI (kg/m^2^), median (IQR)	28.3 (25.8–30.4)	28.4 (26.4–30.2)	0.371
Hypertension, % (*n*)	65.6 (145)	65.5 (133)	1.000
Diabetes, % (*n*)	43.9 (97)	42.4 (86)	0.827
COPD, % (*n*)	27.1 (60)	23.6 (48)	0.474
Hyperlipidemia, % (*n*)	41.2 (91)	42.9 (87)	0.801
EuroSCORE II, mean ± SD	4.33 ± 2.04	3.97 ± 2.25	0.080

BMI, body mass index; COPD, chronic obstructive pulmonary disease; IQR, interquartile range; SD, standard deviation; TCRAT, total coronary revascularization via left anterior thoracotomy; MS, median sternotomy.

Preoperative laboratory findings showed no significant differences between the two groups. An echocardiographic assessment revealed similar LVEF, while the left atrial diameter and SPAP were notably higher in the MS group (Table 2).

**Table 2:** Laboratory and echocardiographic findings.

Variable	TCRAT (*n* = 221)	MS (*n* = 203)	*p*-value
WBC count (×10^3^/mm^3^), median (IQR)	7.80 (6.20–9.70)	8.10 (6.30–9.20)	0.815
Hemoglobin (g/dL), median (IQR)	13.50 (12.40–14.40)	13.20 (11.90–14.40)	0.051
Hematocrit (%), median (IQR)	40.80 (37.50–43.70)	40.20 (36.50–44.40)	0.329
Urea (mg/dL), median (IQR)	35.00 (27.00–46.00)	32.00 (24.00–66.40)	0.991
Creatinine (mg/dL), median (IQR)	1.01 (0.87–1.14)	0.98 (0.84–1.19)	0.954
Sodium (mmol/L), median (IQR)	138.00 (135.00–140.00)	138.00 (135.60–141.00)	0.052
eGFR (mL/min/1.73 m^2^), median (IQR)	74.6 (59.1–88.8)	72.6 (56.8–88.2)	0.423
Potassium (mmol/L), median (IQR)	4.10 (3.80–4.40)	4.10 (3.70–4.40)	0.208
Calcium (mmol/L), median (IQR)	9.00 (8.80–9.40)	9.00 (8.60–9.40)	0.158
Magnesium (mmol/L), median (IQR)	2.05 (1.90–2.20)	2.00 (1.80–2.20)	0.155
LDL-C (mg/dL), median (IQR)	124.00 (96.00–151.00)	125.30 (105.00–157.00)	0.127
Total cholesterol (mg/dL), median (IQR)	195.00 (166.00–229.00)	200.00 (174.00–241.50)	0.096
LVEF (%), median (IQR)	55.00 (50.00–55.00)	55.00 (45.00–60.00)	0.894
Left atrium (mm), median (IQR)	35.00 (32.00–38.00)	36.00 (34.00–40.00)	0.001
SPAP (mmHg), median (IQR)	20.00 (15.00–25.00)	25.00 (20.00–30.00)	0.022

TCRAT, total coronary revascularization via left anterior thoracotomy; MS, median sternotomy; IQR, interquartile range; LDL-C, low-density lipoprotein cholesterol; WBC, white blood cell; LVEF, left ventricular ejection fraction, SPAP, systolic pulmonary artery pressure; eGFR, estimated glomerular filtration rate.

Preoperative medication use was consistent across both groups (Table 3).

**Table 3:** Preoperative medications.

Variable	TCRAT (*n* = 221)	MS (*n* = 203)	*p*-value
ACE inhibitor, % (*n*)	45.7 (101)	38.2 (58)	0.180
Beta blocker, % (*n*)	62.0 (137)	52.9 (81)	0.101
ARBs, % (*n*)	24.4 (54)	26.8 (41)	0.693
Calcium channel blockers, % (*n*)	38.0 (84)	34.6 (53)	0.578
Clopidogrel, % (*n*)	24.4 (54)	26.8 (41)	0.693
Statins, % (*n*)	44.3 (98)	42.5 (65)	0.802
Nitrates, % (*n*)	30.3 (67)	37.3 (57)	0.197
Furosemide, % (*n*)	22.2 (49)	16.3 (25)	0.208
Spironolactone, % (*n*)	13.1 (29)	17.0 (26)	0.373
Fibrates, % (*n*)	5.4 (12)	8.5 (13)	0.339
Acetylsalicylic acid, % (*n*)	72.9 (161)	69.9 (107)	0.618

ACE, angiotensin-converting enzyme; ARBs, angiotensin receptor blockers; TCRAT, total coronary revascularization via left anterior thoracotomy; MS, median sternotomy.

Operative data indicated that operation, CPB, and cross-clamp times were significantly longer in the TCRAT group (Table 4). Conversely, blood transfusion requirements were significantly higher in the MS group. ICU and total hospital stays were shorter for the TCRAT group. The incidence of POAF was lower in the TCRAT group than in the MS group (16.7% vs. 25.1%, *p* = 0.045). There were no significant differences in 30-day mortality or stroke rates between the two groups (Table 4).

**Table 4:** Operative and postoperative outcomes.

Variable	TCRAT (*n* = 221)	MS (*n* = 203)	*p*-value
Operation duration (min), median (IQR)	256 (208–322)	213 (183–258)	<0.001
CPB time (min), median (IQR)	159 (124–182)	108 (92.5–132)	<0.001
Cross-clamp time (min), median (IQR)	82 (59–99)	62 (51.5–71.5)	<0.001
Number of grafts, mean ± SD	2.75 ± 1.05	3.03 ± 0.95	0.004
1 graft, % (*n*)	15.4 (34)	6.4 (13)	0.003
2 grafts, % (*n*)	21.3 (47)	15.8 (32)	0.170
≥3 grafts, % (*n*)	63.3 (140)	77.8 (158)	0.002
Use of LIMA graft, % (*n*)	80.1 (177)	88.7 (180)	0.022
Blood transfusion, mean ± SD	1.81 ± 1.15	2.99 ± 1.20	<0.001
Reexploration for bleeding, % (*n*)	3.2 (7)	3.9 (8)	0.867
Length of hospital stay (days), median (IQR)	5 (3–14)	6 (3–14)	<0.001
Length of ICU stay (h), median (IQR)	38 (25–56)	40 (29–72)	0.040
Postoperative atrial fibrillation, % (*n*)	16.7 (37)	25.1 (51)	0.045
30-day mortality, % (*n*)	3.6 (8)	3.9 (8)	1.000
30-day stroke, % (*n*)	3.6 (8)	2.5 (5)	0.683

CPB, cardiopulmonary bypass; ICU, intensive care unit; LIMA, left internal mammary artery; SD, standard deviation; TCRAT, total coronary revascularization via left anterior thoracotomy; MS, median sternotomy.

**Figure 2:**
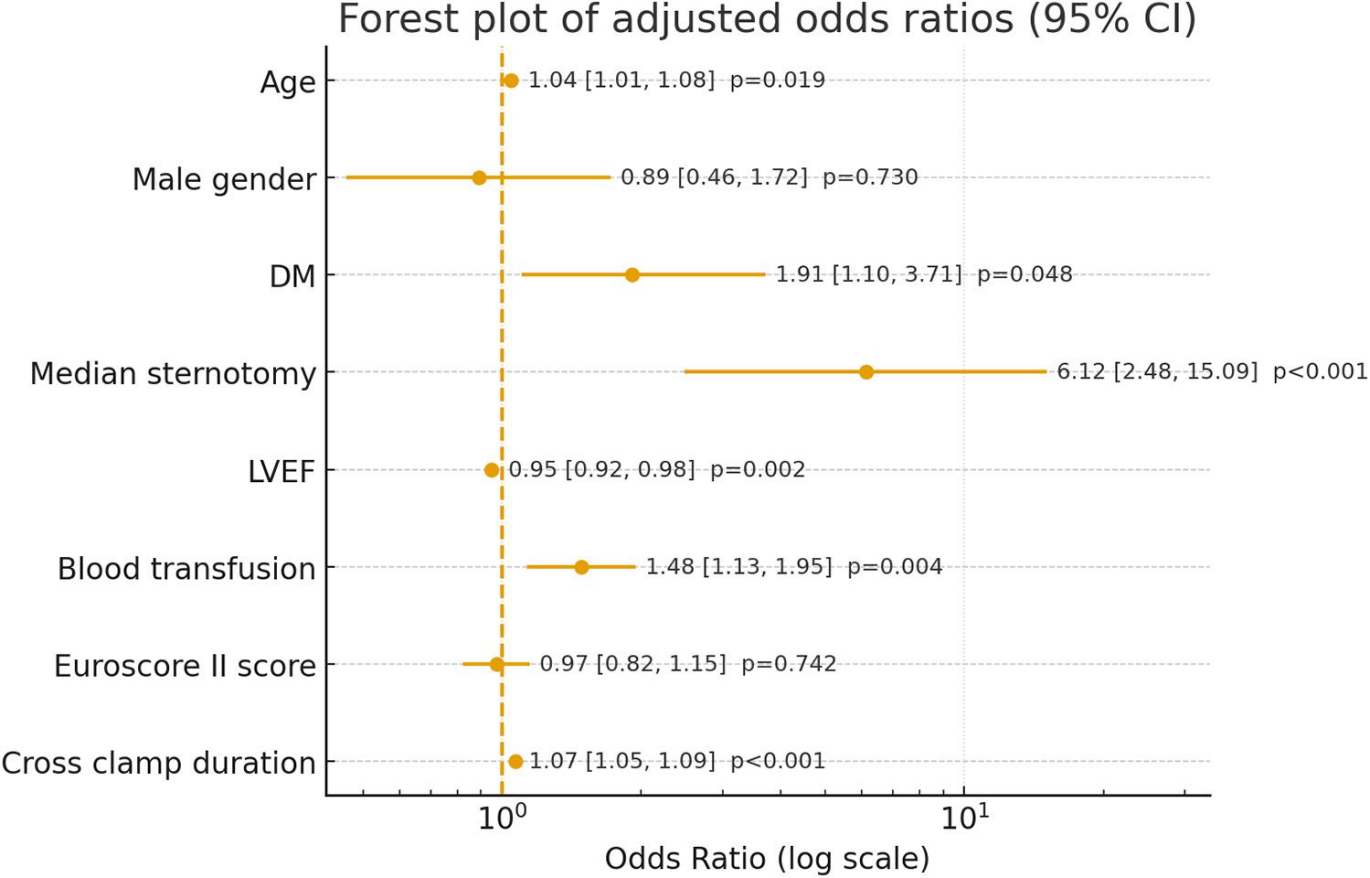
Multivariable logistic regression analysis for predictors of POAF (PSM cohort). LVEF, left ventricular ejection fraction; DM, diabetes mellitus; PSM, propensity score matching; POAF, postoperative atrial fibrillation; TCRAT, total coronary revascularization via left anterior thoracotomy; MS, median sternotomy.

After PSM, baseline characteristics were well balanced (Figure 1). In the multivariable logistic regression analysis, the two types of surgical approaches (MS vs. TCRAT) remained a strong independent predictor of POAF [odds ratio (OR) 6.12; 95% confidence interval (CI), 2.48–15.09; *p* < 0.001]. Other independent predictors included older age, longer cross-clamp time, higher blood transfusion requirements, diabetes, and reduced LVEF (Figure 2, Supplementary Table S3).

## 4 Discussion

Minimally invasive surgical approaches have recently become increasingly common in CABG. Among these, TCRAT is notable for its ability to achieve full grafting of all target vessels (7, 8). While ongoing studies continue to assess the efficacy and safety of TCRAT (11, 12), its impact on arrhythmic complications remains unclear. The learning curve associated with this technique is also significant. Babliak et al. reported that operative times decreased after approximately the 30th case, with no major improvements thereafter (7). Therefore, in this study, we included only those patients who were operated upon after each surgeon's 30th TCRAT case in order to reduce the influence of the learning curve.

The rate of incidence of POAF following CABG performed via MS has been reported in the literature to be approximately 20% (2). In our study, the overall POAF rate was 21%; however, the incidence rate was significantly lower in the TCRAT group than in the MS group (16.7% vs. 25.1%). This finding supports the protective effect of minimally invasive approaches against POAF.

The pathophysiology of AF following cardiac surgery differs significantly from that of AF developing in other clinical situations. Factors such as right atriotomy, venous cannulation, myocardial ischemia, reperfusion injury, inflammation, pericardial effusion, and epicardial adipose tissue have been proposed to contribute to its development (13–20). In conventional CABG, right atriotomy performed for central venous cannulation may increase atrial trauma. However, in our study, no right atriotomy was performed in the TCRAT group, and venous cannulation was achieved through the femoral route. This approach may have reduced atrial irritation and inflammatory activation, potentially contributing to the lower incidence of POAF observed. These mechanistic insights should be considered hypothesis-generating rather than definitive causal inferences.

CABG performed with CPB offers certain advantages over off-pump surgery. Patients undergoing on-pump CABG have been reported to achieve higher graft patency, more consistently reach planned graft numbers, and show higher rates of complete revascularization (21). However, the risk of POAF has also been shown to increase in patients undergoing CPB (22), probably because of CPB-induced myocardial ischemia and inflammation. Ascione et al. (23) demonstrated that the levels of neutrophil elastase, IL-8, C3a, and C5a increased during CPB. Aortic cross-clamping, surgical manipulations, and blood contact with artificial surfaces are the primary triggers of the inflammatory response, which is further amplified by macrophage and neutrophil activation, cytokine release, and complement activation.

Patient characteristics also play a crucial role in the development of POAF. With advancing age, structural changes such as atrial dilation, muscle atrophy, fibrosis and adipose infiltration of the sinus node, and local amyloid deposition in the atria increase susceptibility to POAF (24). Mathew et al. (25) demonstrated that the incidence of POAF rose significantly in patients over 70 years old. Similarly, in patients with heart failure, elevated serum aldosterone levels contribute to myocardial fibrosis and prolong atrial repolarization, thereby facilitating conduction disturbances and promoting arrhythmias through reentry mechanisms (26). Velioglu et al. (27) further reported that elevated preoperative SPAP was significantly associated with POAF in off-pump CABG patients. In our study, the higher SPAP and larger left atrial diameter observed in the MS group support the contribution of traditional risk factors to the development of POAF in this group (3).

In our multivariate analysis, the traditional MS surgical approach was identified as a strong independent predictor of POAF. Advanced age, reduced LVEF, prolonged cross-clamp time, increased transfusion requirements, and diabetes also emerged as independent predictors. These findings are consistent with previously established risk factors for POAF and further highlight the role of comorbidities and traditional risk factors in increasing susceptibility to arrhythmias (3).

A notable observation was that, despite longer operative and cross-clamp times in the TCRAT group of patients, the incidence of POAF was lower in them. This suggests that the potential advantages of the minimally invasive approach (e.g., reduced pericardial and atrial manipulation, and reduced blood loss) outweigh the negative effects of prolonged operative time. Reduced trauma to the chest wall and pericardium, lower blood loss and transfusion requirements, and consequently, a diminished inflammatory response explain this observation.

The independent association between red blood cell (RBC) transfusion and POAF observed in our study supports the hypothesis that inflammatory and hematologic stress may play a mechanistic role in arrhythmia development. RBC transfusions can exert proinflammatory effects because of changes that occur during processing and storage. The release of free iron, microparticle emission, and storage lesions from erythrocytes is known to enhance endothelial activation and amplify inflammatory responses (28). Moreover, it has been shown that RBC transfusion increases the circulating levels of von Willebrand factor, indicating endothelial activation independent of sepsis or organ injury (29). This observation aligns with the hypothesis that inflammatory cascades and endothelial dysfunction contribute to POAF but should be interpreted as hypothesis-generating rather than conclusive evidence.

In our study, the lower incidence of POAF was consistent with shorter ICU and hospital stays and indicate a potential to reduce the long-term risk of stroke and mortality. Conversely, the similar 30-day mortality and stroke rates between the two groups of patients suggested that rhythm-related benefits did not compromise short-term safety.

### 4.1 Limitations

This study has several limitations. First, because it was retrospective and based on patient records, the collection of data on some factors such as preoperative medications, lifestyle habits, and comorbidities depended on patient self-reporting perhaps rendering the data incomplete or inaccurate.

Second, postoperative rhythm monitoring was based on continuous observation and standard ECG recordings during hospitalization, without the use of Holter or extended monitoring systems. Consequently, short or asymptomatic AF episodes might have gone undetected. The exact timing of POAF onset was not consistently documented, preventing a sensitivity analysis limited to the ICU or first 48 h. In addition, the shorter hospital stays of patients of the TCRAT group may have further prevented the detection of late POAF.

Third, echocardiographic assessments were conducted by different cardiologists across centers, which could have introduced variability in measurements such as left ventricular function and atrial size.

Fourth, because of the limited sample size, sensitivity analyses based on graft number or type (≥3 vs. 1–2) and conditional logistic regression could not be performed within the current matching framework.

Fifth, although early learning curve cases were excluded, residual bias related to operator experience and calendar time could not be completely eliminated.

Finally, the relatively short study period (2022–2024) and the moderate sample size limited the generalizability of the findings. Long-term outcomes such as graft patency, late AF development, and survival could not be assessed. Although propensity score matching was used, residual confounding from unmeasured variables cannot be completely ruled out.

In summary, while our study provides meaningful insights into predictors of POAF, further confirmation of these insights through larger, prospective, multicenter studies with longer follow-up is warranted.

## 5 Conclusion

In this multicenter retrospective study, it was found that the incidence of POAF was significantly lower in patients who underwent surgery in which the TCRAT technique was used compared with those treated with MS, the traditional surgical approach. MS, advanced age, reduced LVEF, prolonged cross-clamp time, diabetes, and increased transfusion requirements were identified as independent risk factors for POAF. These findings suggest that TCRAT is a safe and effective approach, potentially offering improved rhythm-related outcomes following CABG.

## Data Availability

The original contributions presented in the study are included in the article/Supplementary Material, further inquiries can be directed to the corresponding author.

## Supplementary material

Supplementary Figure S1 Visualization of missing data proportions and imputation diagnostics for baseline and echocardiographic variables.

Supplementary Figure S2 Calibration plot of the multivariable logistic regression model for predicting POAF. Observed event rates are plotted against predicted probabilities, demonstrating good agreement between predicted and observed outcomes (Hosmer–Lemeshow *p* = 0.510).
