# Supplementary figures and images for: Comparison of postoperative atrial fibrillation after total coronary revascularization via left anterior thoracotomy and conventional median sternotomy coronary artery bypass grafting
Source: Front Cardiovasc Med. 2025 Oct 31;12:1697113. doi: 10.3389/fcvm.2025.1697113 (PMC12615368; doi:10.3389/fcvm.2025.1697113)

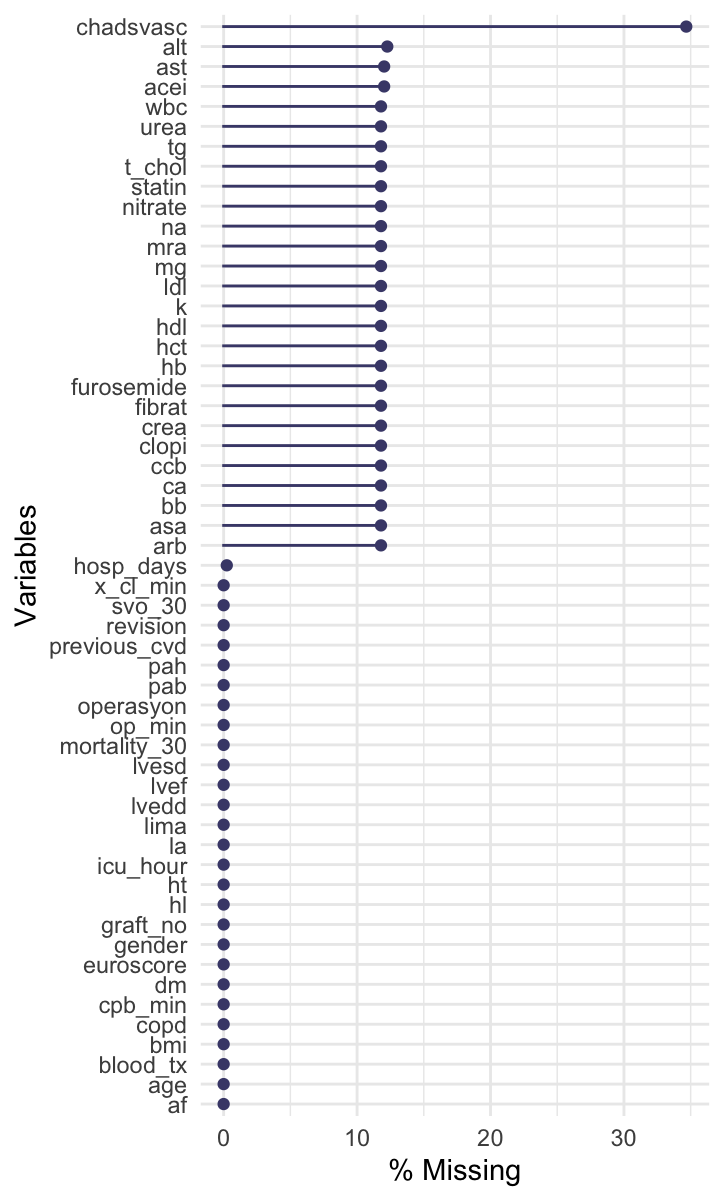

Supplement: Supplementary Figure S1 — Visualization of missing data proportions and imputation diagnostics for baseline and echocardiographic variables. [file Image1.tiff]
