# Supplementary material for: Comparison of postoperative atrial fibrillation after total coronary revascularization via left anterior thoracotomy and conventional median sternotomy coronary artery bypass grafting
Source: Front Cardiovasc Med. 2025 Oct 31;12:1697113. doi: 10.3389/fcvm.2025.1697113 (PMC12615368; doi:10.3389/fcvm.2025.1697113)
Supplement: Supplementary file 3 [file Table1.docx]

| **Supplementary Table S1. Balance diagnostics for matched and non-matched variables** | | | | |
| --- | --- | --- | --- | --- |
|  | Pre-matching SMD | Post-matching SMD | Pre-matching variance ratio | Post-matching variance ratio |
| Number of grafts | 0.297 | 0.450 | 0.828 | 0.834 |
| SPAP | 0.220 | -0.019 | 1.383 | 0.995 |
| CHADSVASC score | -0.286 | -0.054 | 1.131 | 1.164 |
| Age | 0.207 | 0.006 | 1.199 | 1.196 |
| LVEF | -0.046 | 0.004 | 1.497 | 1.609 |
| Gender | 0.001 | 0.013 | 1.000 | 1.000 |
| Left atrial diameter | 0.269 | -0.061 | 0.793 | 0.641 |
| Preop BB | -0.101 | 0.009 | NA | NA |

(SPAP: systolic pulmonary artery pressure; **SMD: Standardized Mean Difference;** LVEF: left ventricular ejection fraction; Preop BB: preoperative β-blocker)
