# Supplementary material for: Comparison of postoperative atrial fibrillation after total coronary revascularization via left anterior thoracotomy and conventional median sternotomy coronary artery bypass grafting
Source: Front Cardiovasc Med. 2025 Oct 31;12:1697113. doi: 10.3389/fcvm.2025.1697113 (PMC12615368; doi:10.3389/fcvm.2025.1697113)
Supplement: Supplementary file 4 [file Table2.docx]

| **Supplementary Table S2: Variation Inflation Factor Levels for Assessing Multicollinearity.** | |
| --- | --- |
| Variable | VIF levels |
| Operation duration | 2.24 |
| Cross-clamp time | 1.92 |
| Age | 1.63 |
| Euroscore II | 1.49 |
| Blood transfusion | 1.37 |
| Diabetes mellitus | 1.25 |
| LVEF | 1.16 |
| Male gender | 1.07 |

**Supplementary Table S2:** Variation Inflation Factor Levels for Assessing Multicollinearity.

(LVEF: left ventricular ejection fraction;)
