# Supplementary material for: Comparison of postoperative atrial fibrillation after total coronary revascularization via left anterior thoracotomy and conventional median sternotomy coronary artery bypass grafting
Source: Front Cardiovasc Med. 2025 Oct 31;12:1697113. doi: 10.3389/fcvm.2025.1697113 (PMC12615368; doi:10.3389/fcvm.2025.1697113)
Supplement: Supplementary file 5 [file Table3.docx]

| **Supplementary Table S3. Final multivariable logistic regression model for predictors of POAF (matched cohort)** | | | |
| --- | --- | --- | --- |
| Variable | Odds Ratio (OR) | 95% Confidence Interval (CI) | p-value |
| Operation type (MS vs TCRAT) | 6.12 | 2.48 – 15.09 | <0.001 |
| Age (per year) | 1.04 | 1.01 – 1.08 | 0.019 |
| LVEF (per %) | 0.95 | 0.92 – 0.98 | 0.002 |
| Diabetes mellitus | 1.91 | 1.10 – 3.71 | 0.048 |
| Cross-clamp time (per 10 min) | 1.07 | 1.05 – 1.09 | <0.001 |
| Blood transfusion (≥1 unit) | 1.48 | 1.13 – 1.95 | 0.004 |
| Euroscore II score | 0.97 | 0.82 – 1.15 | 0.742 |
| Male gender | 0.89 | 0.46 – 1.72 | 0.730 |

Model performance: Hosmer–Lemeshow χ² = 6.02 (df = 8, p = 0.510); AUC = 0.79. No significant multicollinearity observed (all VIF < 2.5).

(LVEF: left ventricular ejection fraction; POAF: postoperative atrial fibrillation; TCRAT: total coronary revascularization via left anterior thoracotomy; MS: median sternotomy)
